# Survival trend and outcome prediction for pediatric Hodgkin and non-Hodgkin lymphomas based on machine learning

**DOI:** 10.1007/s10238-024-01402-3

**Published:** 2024-06-18

**Authors:** Yue Zheng, Chunlan Zhang, Xu Sun, Kai Kang, Ren Luo, Ailin Zhao, Yijun Wu

**Affiliations:** 1 Division of Thoracic Tumor Multimodality Treatment, Cancer Center, West China Hospital, Sichuan University, Chengdu, China; 2 Laboratory of Clinical Cell Therapy, West China Hospital, Sichuan University, Chengdu, China; 3 Department of Hematology, West China Hospital, Sichuan University, Chengdu, China

**Keywords:** Pediatric lymphoma, Survival trend, Outcome prediction, Nomogram, Machine learning

## Abstract

Pediatric Hodgkin and non-Hodgkin lymphomas differ from adult cases in biology and management, yet there is a lack of survival analysis tailored to pediatric lymphoma. We analyzed lymphoma data from 1975 to 2018, comparing survival trends between 7,871 pediatric and 226,211 adult patients, identified key risk factors for pediatric lymphoma survival, developed a predictive nomogram, and utilized machine learning to predict long-term lymphoma-specific mortality risk. Between 1975 and 2018, we observed substantial increases in 1-year (19.3%), 5-year (41.9%), and 10-year (48.8%) overall survival rates in pediatric patients with lymphoma. Prognostic factors such as age, sex, race, Ann Arbor stage, lymphoma subtypes, and radiotherapy were incorporated into the nomogram. The nomogram exhibited excellent predictive performance with area under the curve (AUC) values of 0.766, 0.724, and 0.703 for one-year, five-year, and ten-year survival, respectively, in the training cohort, and AUC values of 0.776, 0.712, and 0.696 in the validation cohort. Importantly, the nomogram outperformed the Ann Arbor staging system in survival prediction. Machine learning models achieved AUC values of approximately 0.75, surpassing the conventional method (AUC =  ~ 0.70) in predicting the risk of lymphoma-specific death. We also observed that pediatric lymphoma survivors had a substantially reduced risk of lymphoma after ten years b,ut faced an increasing risk of non-lymphoma diseases. The study highlights substantial improvements in pediatric lymphoma survival, offers reliable predictive tools, and underscores the importance of long-term monitoring for non-lymphoma health issues in pediatric patients.

**Supplementary Information:**

The online version contains supplementary material available at 10.1007/s10238-024-01402-3.

## Introduction

Lymphoma stands as the third most prevalent pediatric cancer, comprising 15% of childhood malignancies [1]. Despite significant advancements in treatment approaches that have markedly improved the outlook for pediatric lymphoma patients in recent decades, lymphoma remains a notable contributor to childhood cancer-related mortality [2, 3]. This is especially true for children aged 1–10 years. Notably, treatment outcomes can exhibit considerable variability, potentially attributed to a complex interplay of psychosocial factors, patient-specific variables, tumor subtypes, and their underlying biological characteristics [4]. Therefore, it is imperative to conduct a comprehensive investigation on a substantial scale to discern the factors influencing survival and prognosis in pediatric lymphoma patients.

Recent investigations have illuminated the shifting landscape of pediatric lymphoma through extensive database analyses. For example, Kahn et al. [5] delved into racial disparities in the survival of pediatric Hodgkin lymphoma (HL) patients, revealing that Black patients exhibited a significantly lower 10-year overall survival (OS) rate compared to Caucasians. Interestingly, this survival gap has been narrowing over time, primarily due to more substantial improvements in the ten-year OS rates observed among Black patients. In a similar vein, Bazzeh et al. [6] focused their exploration on pediatric HL patients spanning from 1988 to 2005, identifying stage IV disease and the presence of B symptoms as independent prognostic risk factors. Various studies have focused on specific facets or subtypes of pediatric non-Hodgkin lymphoma (NHL), such as cutaneous T-cell or B-cell lymphoma, as well as primary gastrointestinal lymphoma within the Surveillance, Epidemiology, and End Results (SEER) database [7–9]. The imperative for a comprehensive investigation into the survival and prognosis of both HL and NHL in pediatric patients remains paramount. Therefore, building upon the extensive clinical data available in the SEER database, encompassing patients with lymphoma from 1975 to 2018, we aimed to comprehensively explore the survival and prognosis predictors of pediatric lymphoma, serving as the foundation for the development of machine learning models capable of reliably predicting survival outcomes. Simultaneously, this study sought to analyze survival trends over recent decades to pinpoint key aspects that could guide the future trajectory of pediatric lymphoma research.

## Subjects and methods

### Data source

The data for this study were sourced from cancer records spanning a period from 1975 to 2018, originating from nine specific states within the United States through SEER database collected and consolidated by the National Cancer Institute as part of its commitment to tackling the increasing burden of cancer. The selected states that contributed to this dataset encompass Connecticut, Michigan, Georgia, California, Hawaii, Iowa, New Mexico, Washington, and Utah. The SEER database, accessible at https://seer.cancer.gov, stands as a comprehensive repository of cancer-related information. A visual representation of the study's flow and methodology can be found in Supplemental Figure S1**.**

### Patient enrollment

Patients diagnosed with primary lymphoma at ages ranging from 0 to 19 years were identified using the third edition of the International Classification of Diseases for Oncology. To conduct survival-associated analyses, additional screening was carried out to exclude cases lacking follow-up information or involving patients who passed away within one month after their diagnosis. Extensive demographic and clinical data pertaining to the patients were meticulously gathered. This encompassed data such as the age at diagnosis, sex, race, tumor subtype, Ann Arbor staging, year of diagnosis, the utilization of chemotherapy and radiotherapy, and vital status. It is important to underscore that the execution of this study was carried out in strict adherence to the Strengthening the Reporting of Observational Studies in Epidemiology guideline, ensuring the robustness and transparency of the research methodology [10].

### Outcome prediction tools

The analysis of factors associated with OS among pediatric lymphoma patients was carried out using a multivariable Cox proportional hazards regression model. A nomogram model, built upon the most influential factors, was developed to predict OS at one-year, five-year, and ten-year intervals. This model underwent external validation within a separate validation cohort, created through random division at a 7:3 ratio. The model's precision was confirmed by assessing the area under the curve (AUC) of receiver operating characteristic (ROC), and comparisons were made between the nomogram and the Ann Arbor staging system. Additionally, a calibration curve was generated to compare the predictive outcomes of the nomogram against actual survival rates.

Five well-established machine learning algorithms were employed to predict the long-term risk of lymphoma-specific mortality. These algorithms included extreme gradient boosting (XGB), the random forest classifier (RFC), adaptive boosting (ADB), artificial neural network (ANN), and gradient boosting decision tree (GBDT), alongside logistic regression (LR). The parameters for each machine learning algorithm are shown in Supplemental Table S1. The ANN algorithm is a complex, highly interconnected network composed of adaptable units that mimic the interaction of biological nervous systems with real-world objects. RFC represents an advanced iteration of the decision tree algorithm, suitable for both regression and classification tasks. GBDT, XGB, and ADB are part of the ensemble learning category of machine learning algorithms, known for improving classifier generalization by training multiple classifiers and combining their results for enhanced predictive performance. Additionally, to enhance the reliability of models, continuous variables underwent *z*-score normalization as preprocessing. Except for LR, the transparency of these algorithms is limited, making it challenging for users to decipher the relationship between variables and outcomes. To enhance the reliability of models, continuous variables underwent z-score normalization, and categorical variables were one-hot encoded. Feature selection using Cox regression identified potential prognostic predictors.

The training procedure involved several key steps. Each algorithm was trained using fivefold cross-validation to ensure robustness and prevent overfitting, with the datasets split in a 7:3 ratio for training and validation. For the ANN model, the Adam optimizer was employed with a binary cross-entropy loss function, trained for 100 epochs with a batch size of 32. Early stopping was implemented to prevent overfitting by monitoring the validation loss and halting training if no improvement was observed for ten consecutive epochs. The performance of each model was evaluated using the area under the curve (AUC) of receiver operating characteristic (ROC) curves, and decision curve analysis (DCA) was conducted to assess clinical utility.

### Statistical analysis

The extraction of patient data, including clinical characteristics and follow-up information, was conducted using SEER*Stat version 8.3.9 software, accessible at https://seer.cancer.gov/seerstat. Subsequent statistical analyses were performed using IBM SPSS version 27.0, headquartered in Armonk, NY, USA, and R software version 4.3.1, available at https://www.r-project.org. To compare baseline characteristics between the training and validation cohorts, the χ2 test was employed, encompassing variables such as gender, race, age, lymphoma subtype, Ann Arbor stage, and the initial treatment course (involving chemotherapy and radiotherapy). These variables were further subjected to multivariable Cox proportional hazards regression analysis, which calculated hazard ratios (HR) and their associated 95% confidence intervals (CI) with respect to OS. Survival curves for both OS and disease-specific survival (DSS, lymphoma-specific) were generated using the Kaplan–Meier method, and distinctions among various subpopulations were assessed via a log-rank test. Within the SEER program, the survival time was defined as the duration from the date of diagnosis to either death or the most recent follow-up. It's important to note that the patient data utilized in this study were most recently updated as of November 2020. In this study, statistical significance was determined using a two-sided *P* value < 0.05.

## Results

### Patient characteristics

A cohort of 7871 pediatric individuals, ranging in age from 0 to 19 years with a median age of 15 years, received diagnoses of lymphoma between 1975 and 2018. These cases were extracted from the SEER database, which draws data from nine U.S. states (Supplemental Table S2). The majority of these patients, constituting 53.6% (*N* = 4215), fell within the 15–19-year age group. Furthermore, 6.5% (*N* = 513) of the cases were in the 0–4-year age group, 14.4% (*N* = 1137) were aged 5–9 years, and 25.5% (*N* = 2006) were aged 10–14 years. Males accounted for a higher proportion, at 59.1% (*N* = 4650), compared to females at 40.9% (*N* = 3221). The ethnic distribution showed that Caucasians comprised the largest group, with 80.5% (*N* = 6338), followed by 11.4% (*N* = 897) who were of African descent, and 8.1% (*N* = 556) from other ethnic backgrounds, including AI/AN/AP (American Indian/Alaska Native/Asian and Pacific Islander). In terms of lymphoma subtypes, 53.3% (*N* = 4193) were diagnosed with HL, of which 4144 were nodal and 49 were extra-nodal, while 46.7% (*N* = 3678) had NHL, with 2543 being nodal and 1135 extra-nodal cases. Among the 5579 (70.9%) cases with staging information available, 17.6% (*N* = 1388) were categorized as stage I, 24.2% (*N* = 1901) as stage II, 11.4% (*N* = 896) as stage III, and 17.7% (*N* = 1394) as stage IV. As of the latest update, 1859 patients (23.6%) have succumbed to the condition, while 5992 (76.1%) remain alive. It's worth noting that 20 patients (0.3%) among the surviving group were lost to follow-up.

### Survival trend analysis

**Fig. 1:**
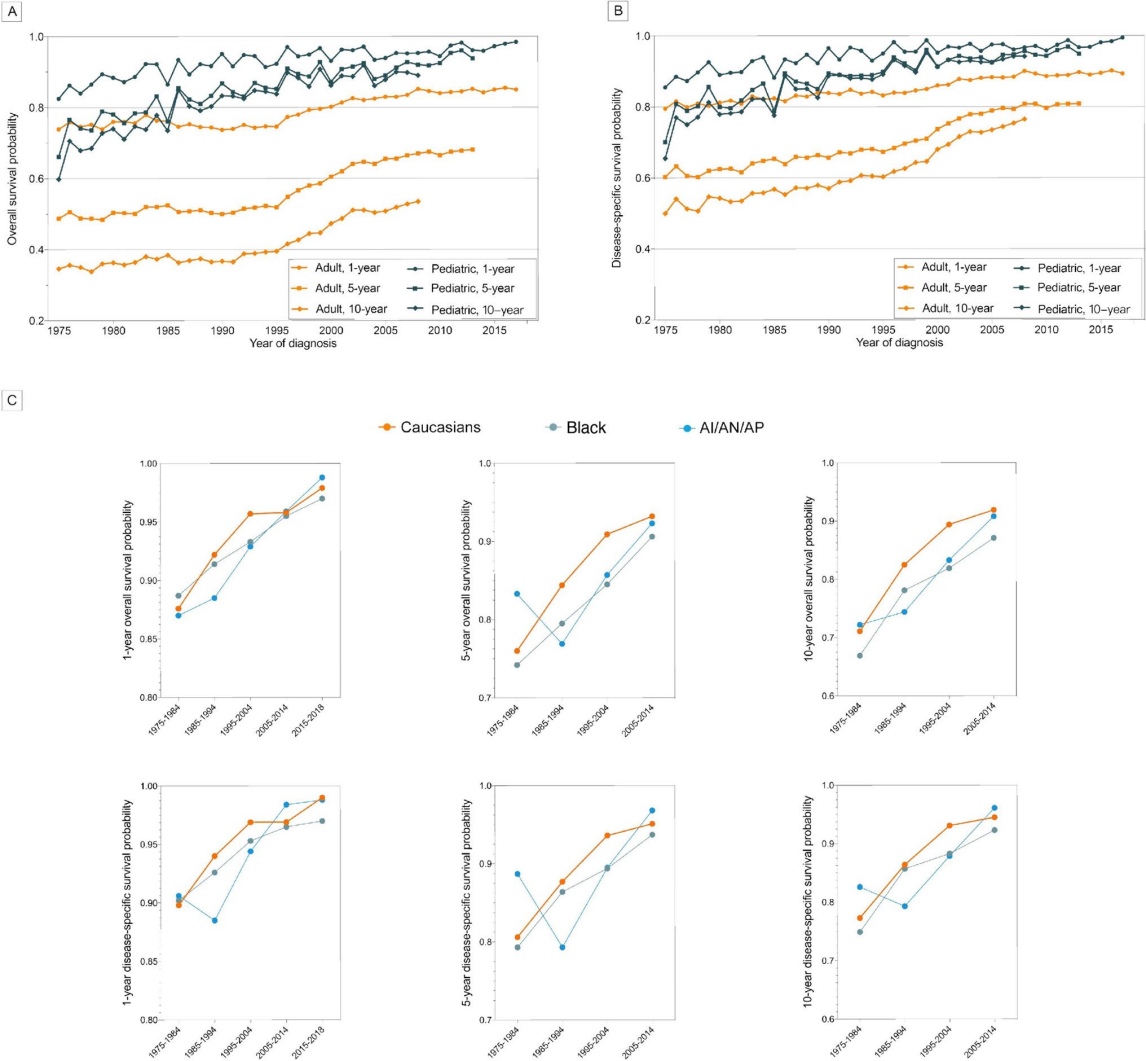
Overall survival and disease-specific survival trends of pediatric lymphoma over time. **A** 1-year, 5-year, and 10-year overall survival rates of pediatric and adult lymphoma over the year of diagnosis. **B** 1-year, 5-year, and 10-year disease-specific survival rates of pediatric and adult lymphoma over the year of diagnosis. **C** 1-year, 5-year, and 10-year overall survival and disease-specific survival rates of pediatric lymphoma patients among the subgroups of different races over the year of diagnosis. aAI/AN/AP, American Indian/Alaska Native/Asian and Pacific Islander

As shown in Fig. 1 and Supplemental Table S2, both adult and pediatric patients with lymphoma demonstrated gradually improved OS and DSS over the past four decades. Among the pediatric patients, the 1-year, 5-year and 10-year OS probability rates increased by 19.3% (82.5% in 1975 to 98.4% in 2017), 41.9% (66.1% in 1975 to 93.8% in 2013), and 48.8% (59.8% in 1975 to 89.0% in 2008), compared with 15.0% (73.9% in 1975 to 85.0% in 2017), 39.8% (48.7% in 1975 to 68.1% in 2013), and 54.6% (34.6% in 1975 to 53.5% in 2008) among adults, respectively. As for DSS, increases in the 1-year, 5-year, and 10-year rates for pediatric patients were 16.3% (85.5% in 1975 to 99.4% in 2017), 35.4% (70.1% in 1975 to 94.9% in 2013), and 43.8% (65.5% in 1975 to 94.2% in 2008), while adult cases were 12.3% (79.5% in 1975 to 89.3% in 2013), 34.2% (60.3% in 1975 to 80.9% in 2013), and 53.0% (50.0% in 1975 to 76.5% in 2008), respectively. Kahn et al. [5] reported that the Black population showed more prominent improvement in the long-term survival than Caucasians. In contrast, our subgroup analyses of different races demonstrated that Caucasian children showed consistently higher survival rates, especially in the 5-year and 10-year outcomes (Fig. 1C).

### Prognostic analysis

**Fig. 2:**
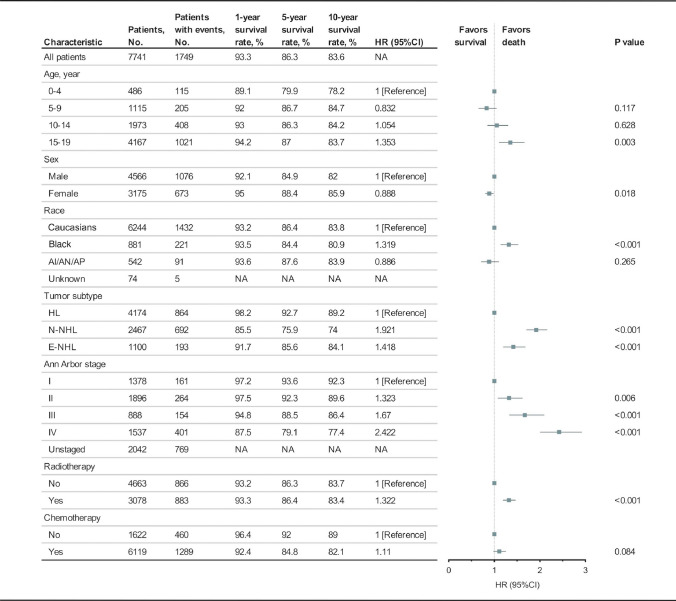
Multivariable Cox proportional hazards regression analysis for overall survival among pediatric patients with lymphoma. aHR, hazard ratio; AI/AN/AP, American Indian/Alaska Native/Asian and Pacific Islander; HL, Hodgkin lymphoma; N-NHL, Nodal Non-Hodgkin lymphoma; E-NHL, extra-nodal non-Hodgkin lymphoma

Using the multivariable Cox regression model, the independent prognostic risk factors for OS among pediatric patients with lymphoma were identified, including age (0–4 years: reference, HR = 1; 5–9 years: HR = 0.83, 95%CI 0.66–1.05, *P* = 0.117; 10–14 years: HR = 1.05, 95%CI 0.85–1.30, *P* = 0.628; 15–19 years: HR = 1.35, 95%CI 1.11–1.66, *P* = 0.003), sex (male: reference, HR = 1; female: HR = 0.89, 95%CI 0.81–0.98, *P* = 0.018), race (Caucasian: reference, HR = 1; Black: HR = 1.32, 95%CI 1.14–1.52, *P* < 0.001), the lymphoma subtype (HL: reference, HR = 1; Nodal NHL: HR = 1.92, 95%CI 1.71–2.16, *P* < 0.001; Extra-nodal NHL: HR = 1.42, 95%CI 1.20–1.69, *P* < 0.001), the Ann Arbor stage (stage I: reference, HR = 1; stage II: HR = 1.32, 95%CI 1.08–1.62, *P* = 0.006; stage III: HR = 1.67, 95%CI 1.33–2.09, *P* < 0.001; stage IV: HR = 2.42, 95%CI 2.01–2.92, *P* < 0.001), and radiotherapy (not receiving: reference, HR = 1; receiving: HR = 1.32, 95%CI 1.19–1.46, *P* < 0.001) (Fig. 2). The survival curves and comparisons associated with OS and DSS among subgroups divided by age, sex, race, the lymphoma subtype, and the Ann Arbor stage are shown in Supplemental Figure S2–S6. Importantly, among the pediatric patients with lymphoma, age significantly affected OS but not DSS (Supplemental Figure S2). In the first 26 years, approximately, children with a diagnosis at 0–4 years of age performed worse than others and pediatric patients aged 15–19 years demonstrated worse long-term OS. Sex was identified as one of the critical factors affecting not only DSS but also OS (Supplemental Figure S3). In terms of long-term outcomes, females consistently demonstrated better DSS than males. Although female patients had significantly better OS, the two groups started to overlap after approximately 20 years, indicating that other factors and other diseases may have affected the long-term survival rather than the lymphoma per se. Furthermore, the differences related to ethnicity are complicated. Pediatric patients of different races demonstrated similar DSS, while Caucasians and AI/AN/AP demonstrated significantly better OS than Black (Supplemental Figure S4). This is much more likely to be associated with multiple socioeconomic factors, instead of internal ethnicity differences. As for lymphoma subtypes, HL always showed better DSS than NHL among pediatric patients. Although, both subtypes may have no effects on long-term survival (Supplemental Figure S5). Surprisingly, we observed that the OS lines intersected at about 34 years after diagnosis. Pediatric patients with HL may be more susceptible to other associated factors or other diseases than pediatric patients with NHL during the long-term survival period.

### Outcome prediction

**Fig. 3:**
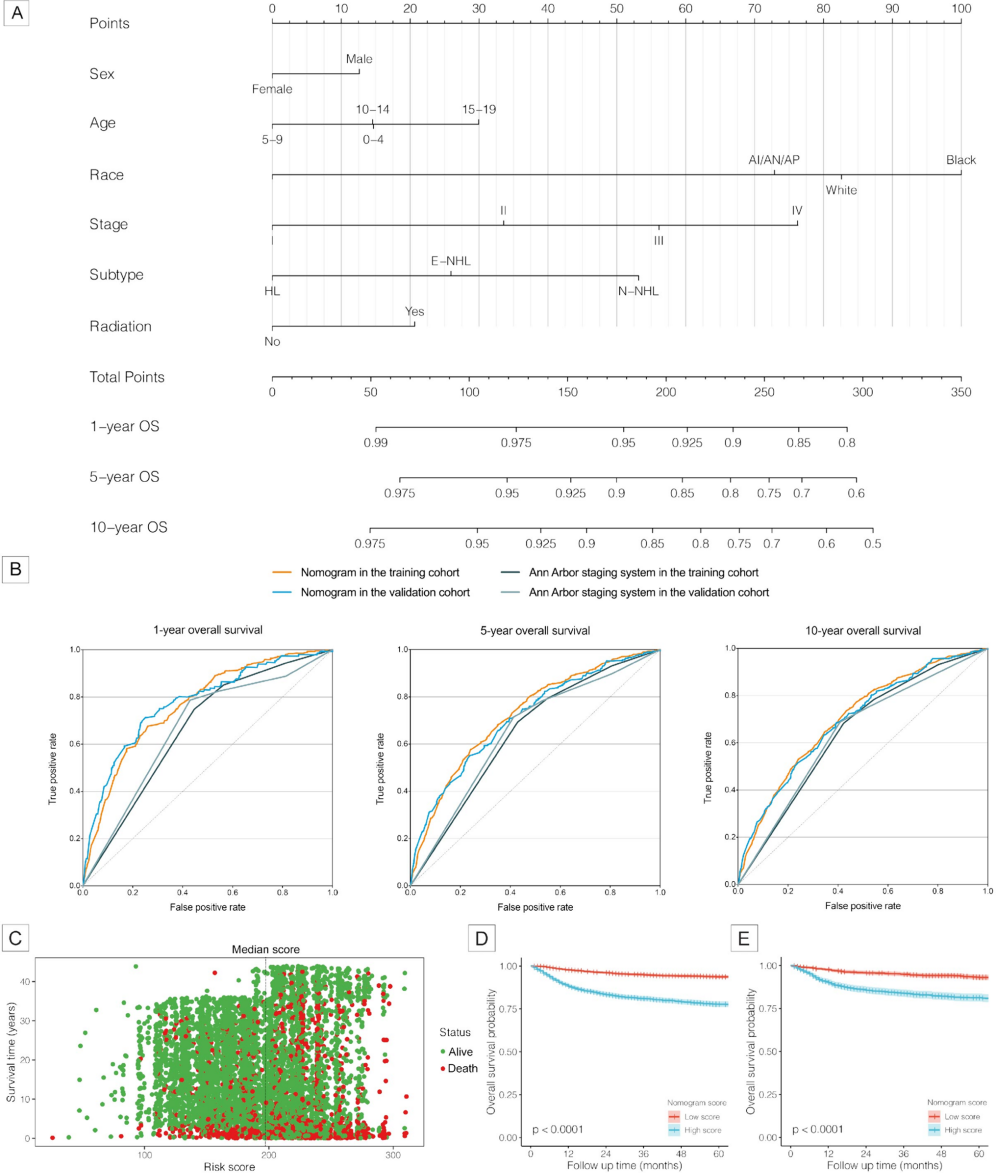
The nomogram to predict 1-year, 5-year, and 10-year overall survival (OS) probabilities among pediatric patients with lymphoma. **A** Quantitative nomogram to predict survival probabilities according to the total points based on sex, age, race, the Ann Arbor stage, the lymphoma subtype, and radiotherapy. White, Caucasians; Black, African-American; AI/AN/AP, American Indian/Alaska Native/Asian and Pacific Islander. **B** Receiver operating characteristic curves of the nomogram and the Ann Arbor Staging System to predict 1-year, 5-year, and 10-year OS probabilities in the training and validation cohorts. AUC, the area under the ROC curve. AUCs of the nomogram (1-year: 0.766 and 0.776, 5-year: 0.724 and 0.712, 10-year: 0.703 and 0.696, in the training and validation cohorts, respectively) vs AUCs of the Ann Arbor Staging System (1-year: 0.666 and 0.668, 5-year: 0.647 and 0.651, 10-year: 0.646 and 0.641, in the training and validation cohorts, respectively). **C** Relationship between nomogram scores and survival time of each pediatric lymphoma patient. **D** and **E** Kaplan–Meier survival curves for pediatric lymphoma patients grouped by the median nomogram score in the training cohort and validation cohort, respectively

A total of 7741 pediatric patients with lymphoma were randomly divided into the training cohort and the validation cohort in a ratio of 7:3. The demographic characteristics of the two cohorts were not significantly different (Supplemental Table S4). Based on the independent prognostic factors identified using the multiple Cox regression model, a prediction nomogram was developed using the variables that involved sex, age, race, Ann Arbor stages, lymphoma subtypes, and radiotherapy in the training cohort (Fig. 3A). Both internal and external validations to test the calibration and predictive ability of the nomogram were performed. Calibration curves of 1-year, 5-year, and 10-year OS demonstrated great consistency between the nomogram-predicted outcomes and the actual OS rates in both the training and the validation cohorts (Supplemental Figure S7). Furthermore, the prediction ability of the nomogram (1-year: 0.766 and 0.776, 5-year: 0.724 and 0.712, 10-year: 0.703 and 0.696, in the training and validation cohorts, respectively) was evaluated by AUCs of the ROC curves, and the nomogram performed better than Ann Arbor staging system (1-year: 0.666 and 0.668, 5-year: 0.647 and 0.651, 10-year: 0.646 and 0.641, in the training and validation cohorts, respectively) (Fig. 3B). We further visualized relationship between all patients’ nomogram scores and survival time (Fig. 3C), and higher nomogram scores indicated significantly worse survival outcomes (Fig. 3D–E).

**Fig. 4:**
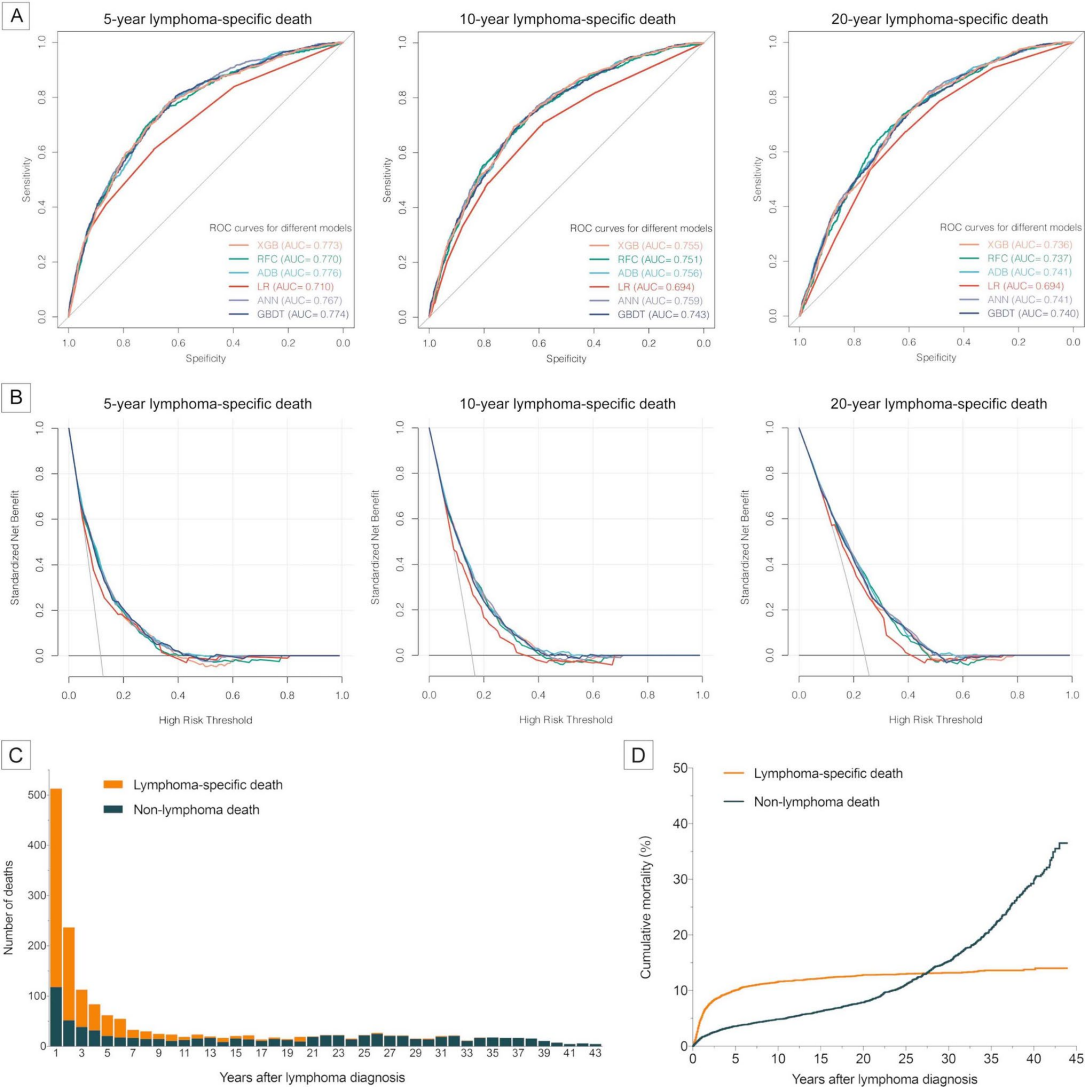
Machine learning models for risk prediction of long-term lymphoma-specific death in patients with pediatric lymphoma. **A** Receiver operating characteristic curves of five classical machine learning-based models and logistic regression (LR) with areas under the curve (AUC). **B** Decision curve analysis for five classical machine learning-based models and LR. **C** Number of lymphoma-specific and non-lymphoma deaths as survival years after lymphoma diagnosis. **D** Cumulative lymphoma-specific and non-lymphoma mortalities as survival years after lymphoma diagnosis

**Fig. 5:**
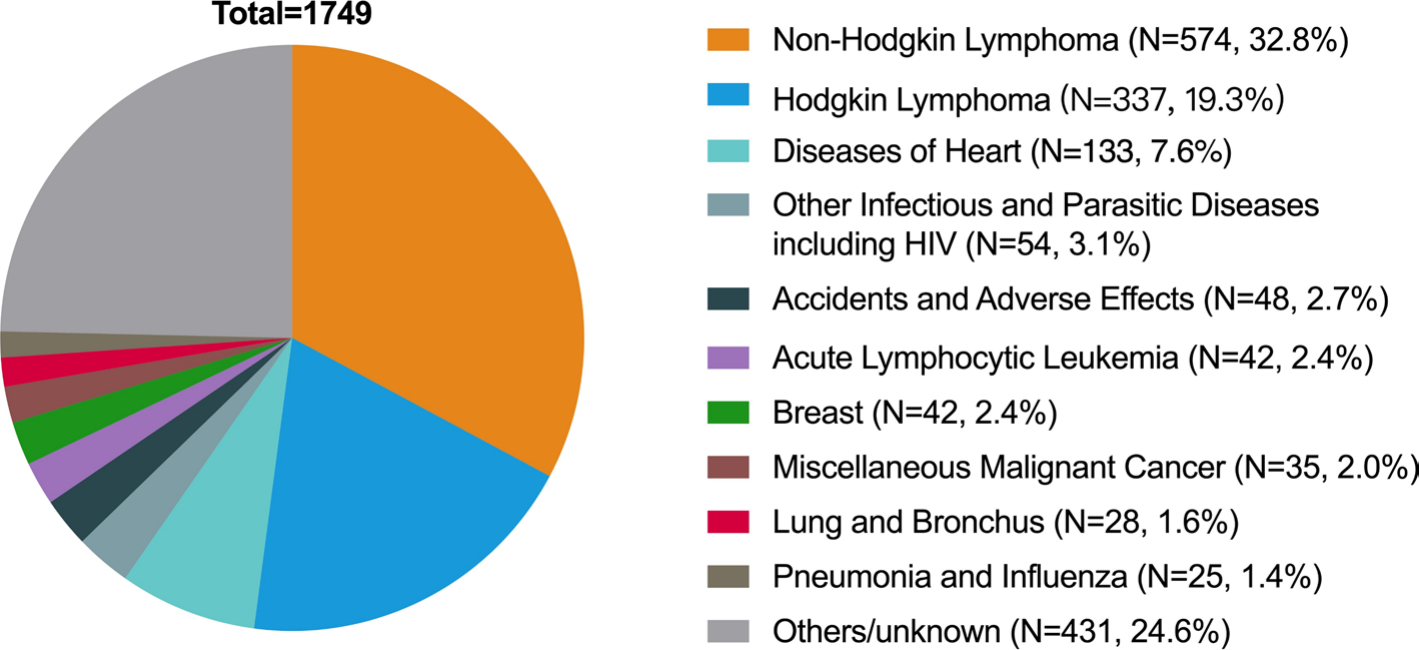
Analysis of death causes among pediatric patients with lymphoma

To further explore relationships between demographic characteristics and long-term outcomes of pediatric lymphoma, we developed multiple machine learning algorithm-based models for predicting the 5-year, 10-year and 20-year risk of lymphoma-specific death using the abovementioned variables. All machine learning models (AUC =  ~ 0.75) demonstrated significantly higher AUCs than conventional LR (AUC =  ~ 0.70) with better performance in decision curves, highlighting the superiority of artificial intelligence (Fig. 4A, B). Furthermore, patients were nearly free from lymphoma-specific death about ten years after diagnosis of pediatric lymphoma, while the non-lymphoma death risk increased sharply all the time (Fig. 4C, D). The sensitivity and specificity values for each model were confirmed at the maximal Youden index (Table 1). The non-lymphoma death causes for pediatric lymphoma patients were shown in Fig. 5.

**Table 1:** Performance of machine learning models for risk prediction of long-term lymphoma-specific death in patients with pediatric lymphoma

Model	Sensitivity (%)	Specificity (%)	AUC	95%CI
5-year lymphoma-specific death risk				
XGB	78.4	63.3	0.773	0.754–0.791
RFC	72.0	69.3	0.770	0.752–0.789
ADB	77.0	64.7	0.776	0.758–0.794
LR	61.0	69.0	0.710	0.699–0.741
ANN	79.3	62.1	0.767	0.749–0.785
GBDT	80.5	60.9	0.774	0.756–0.791
10-year lymphoma-specific death risk				
XGB	69.6	68.9	0.755	0.737–0.772
RFC	73.2	64.0	0.751	0.733–0.769
ADB	71.6	66.3	0.756	0.734–0.773
LR	70.9	58.4	0.694	0.674–0.715
ANN	77.0	60.7	0.759	0.742–0.776
GBDT	72.9	64.7	0.743	0.725–0.761
20-year lymphoma-specific death risk				
XGB	72.6	63.0	0.736	0.718–0.754
RFC	73.5	62.5	0.737	0.719–0.755
ADB	74.4	60.9	0.741	0.723–0.760
LR	67.3	61.4	0.694	0.674–0.713
ANN	74.0	61.4	0.741	0.723–0.759
GBDT	76.3	58.3	0.740	0.722–0.758

AUC: areas under the curve. LR, logistic regression

## Discussion

In this comprehensive population-based study, we leveraged the largest available dataset of cancer patients from the SEER database to conduct a systematic analysis of survival and outcome prediction for pediatric lymphomas, employing advanced machine learning techniques. Our investigation into survival trends revealed a notable increase in OS and DSS over the decades, both in the pediatric and adult lymphoma patient populations. Crucially, our findings indicated a remarkable similarity between 5-year and 10-year survival rates among pediatric patients, implying that the 5-year mark might serve as a critical management checkpoint for long-term survival prospects. It suggests that once pediatric patients with lymphoma surpass the initial 5-year survival threshold, their chances of being cured and enjoying sustained remission significantly improve. Additionally, we observed that OS rates closely mirrored DSS rates within the pediatric population, in stark contrast to the adult population. This intriguing pattern could be attributed to the fact that pediatric patients rarely succumbed to NHL, as evidenced by our analysis of causes of death. Specifically, among deceased pediatric patients, only 52.1% were attributed to lymphoma-related causes (NHL: *N* = 574, 32.8%; Hodgkin Lymphoma: *N* = 337, 19.3%), while other non-lymphoma causes included heart diseases, infectious diseases, accidents, adverse effects, and acute lymphocytic leukemia, among others. Patients with lymphoma were found to have long-term death risk of cardiovascular diseases [11, 12]. The potential immune deficiency caused by lymphomas may also increase the risk of infection [13]. These insights shed light on the complex interplay of factors affecting survival in pediatric lymphoma patients, emphasizing the importance of long-term follow-up and tailored management strategies.

We also performed a multivariable analysis using Cox proportional hazards regression to identify potential independent risk factors for survival outcomes among pediatric patients with lymphoma. Age, sex, race, the lymphoma subtype, the stage, and radiotherapy were found to be significantly associated with OS. The Kaplan–Meier curves also suggested similar survival comparisons. Pediatric patients aged 0–4 years had lower OS than other ages in the first 20 plus years. Surprisingly, we found that the older pediatric patients, aged 15–19 years, demonstrated worse long-term OS outcomes, which was not observed in DSS curves. Consistent with a previous report, in some populations, the number of patients who died of other factors or other diseases can be comparable to those who died of lymphoma itself [14]/Moreover, the cumulative mortality curve also demonstrated that patients after diagnosis of pediatric lymphoma could be exempted from lymphoma-specific death but had an increasing risk of non-lymphoma diseases, especially after surviving ten years. Regardless of OS or DSS, pediatric males had significantly worse survival outcomes than pediatric females. Yet, there was still overlap between the two groups in the OS curve after a follow-up period of more than 30 years. The same situation was also observed between HL and NHL. Our results showed that males and pediatric patients with HL may be more susceptible to some long-term events, such as secondary malignancies and cardiovascular diseases [14–16]. As for race, Caucasian and AI/AN/AP children had significantly better OS than the Black children, while all of them demonstrated similar DSS. Populations of different ethnicities may have specific internal sensitivity to treatment, such as chemotherapy and radiotherapy. Moreover, socioeconomic limitations may lead to delayed diagnoses and management among Black. Furthermore, transplantation is currently one of the most critical treatments for cure, However, Black is under-represented in the marrow donor registries, and thus, have fewer opportunities to undergo transplantation [5].

Though lymphoma does not occur as commonly in children as in adults, it is still one of the most common malignancies among children [17]. The predictive tools that previous studies developed mainly focused on adult lymphoma, very few studies have reported the survival prediction nomogram among pediatric patients with lymphoma. Of note, the Ann Arbor staging system, which focused on the distribution of nodal involvement, was initially developed for HL. The biological features of NHL are different from those of HL. Thus, by integrating the independent prognostic risk factors using one of the largest lymphoma datasets from the SEER database, we developed a predictive nomogram model that can be easily used by clinicians worldwide. We also compared the predictive ability between the nomogram we developed and the Ann Arbor staging system. We found that the nomogram performed better in predicting 1-year, 5-year, and 10-year OS in both the training and validation cohorts. Besides, all machine learning models we developed also performed better than the conventional method in predicting long-term lymphoma-specific death risk, showing the superiority of machine learning in data mining. machine learning models can process large volumes of patient data, including clinical records, images, and genetic information, to assist physicians in devising more personalized treatment plans. In detail, our machine learning models may aid doctors in initial screening and diagnosis, saving time and allowing doctors to focus more on interacting with patients and formulating treatment plans. However, it's important to note that the application of machine learning models requires high-quality data and appropriate regulation to ensure their safety and effectiveness [18]. Additionally, machine learning models should only serve as an auxiliary tool in medical decision-making, with the ultimate treatment decisions still being made by experienced physicians. The ongoing development and improvement of more artificial intelligence tools will contribute to enhancing the diagnosis and treatment outcomes for lymphoma patients [19]. Overall, the quantitative nomogram and machine learning models may be useful for accurately and effectively predicting the survival probability for each individual child and contribute to clinical decision-making.

Our study had several limitations that merit consideration. Firstly, the clinical data we relied upon were sourced from the SEER database, representing only nine U.S. states. As a result, it is essential to acknowledge that our findings might not be entirely representative of the entire pediatric lymphoma landscape across the United States. Nevertheless, it is noteworthy that our study boasted the largest cohort of pediatric lymphoma patients to date, enabling systematic clinical analyses. Another limitation pertains to the comprehensiveness of information within the SEER database. Certain critical clinical details, such as specific treatment protocols, were regrettably unavailable, limiting the further optimization for enhanced predictive accuracy. To address these limitations and enhance the precision of our predictive models, our future plans include launching a multicenter cohort study that encompasses a broader array of pediatric lymphoma patients, thus allowing for the collection of a more comprehensive dataset encompassing a wider range of patient characteristics.

In conclusion, our study pioneered the exploration of survival trends, revealing that advancements in diagnostic and treatment approaches have led to notable improvements in both short-term and long-term survival outcomes. Moreover, we introduced an innovative quantitative nomogram and deployed multiple machine learning models to facilitate outcome prediction, showcasing their remarkable predictive accuracy and practical utility. The insights gleaned from this comprehensive clinical investigation are poised to offer valuable and actionable information on pediatric lymphoma, benefitting clinicians globally and serving as a catalyst for further research in this field.

## Supplementary Information

Below is the link to the electronic supplementary material.

Supplementary file1 (PDF 983 kb)

## Data Availability

The data is available on the Surveillance, Epidemiology, and End Results (SEER, http://seer.cancer.gov) database.
