# Supplementary material for: Survival trend and outcome prediction for pediatric Hodgkin and non-Hodgkin lymphomas based on machine learning
Source: Clin Exp Med. 2024 Jun 18;24(1):132. doi: 10.1007/s10238-024-01402-3 (PMC11189314; doi:10.1007/s10238-024-01402-3)
Supplement: Supplementary file 1 — Supplementary file1 (PDF 983 kb) [file 10238_2024_1402_MOESM1_ESM.pdf]

Survival trend and outcome prediction for pediatric Hodgkin and non-Hodgkin lymphomas based on machine learning

Yue Zheng[1,2,†], Chunlan Zhang[3,†], Xu Sun[3], Kai Kang[1,2], Ren Luo[1,2], Ailin Zhao[3,*] and Yijun Wu[1,2,*]

[1]Division of Thoracic Tumor Multimodality Treatment, Cancer Center, West China Hospital, Sichuan University, Chengdu, China

[2]Laboratory of Clinical Cell Therapy, West China Hospital, Sichuan University, Chengdu, China

[3]Department of Hematology, West China Hospital, Sichuan University, Chengdu, China

[†]Yue Zheng and Chunlan Zhang contributed equally to this work.

***** Correspondence:

Ailin Zhao, MD; E-mail: irenez20@outlook.com

Department of Hematology, West China Hospital, Sichuan University, Chengdu, China

Yijun Wu, MD; E-mail: wuyj01029@wchscu.cn

Division of Thoracic Tumor Multimodality Treatment, Cancer Center, West China Hospital, Sichuan University, Chengdu, China

Figure S1. Study flowchart

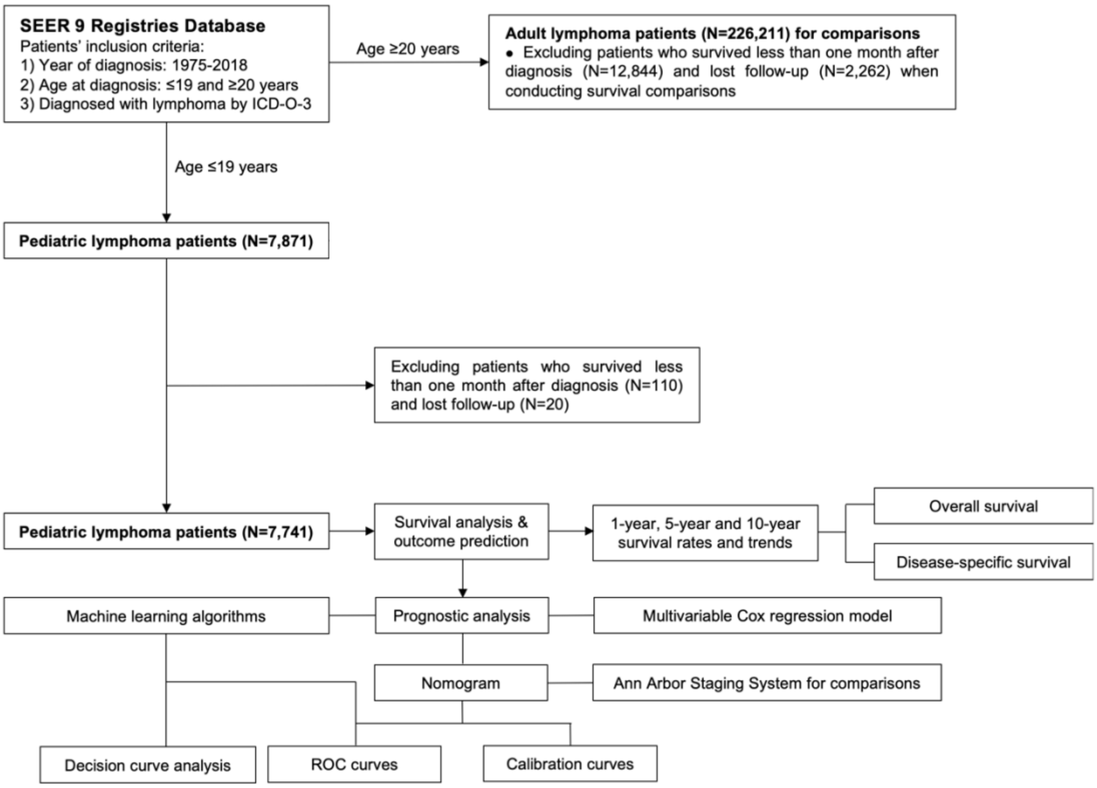

Figure S2. Kaplan–Meier curves for the overall survival and disease-specific survival comparisons between pediatric lymphoma patients of different ages (1975-2018)

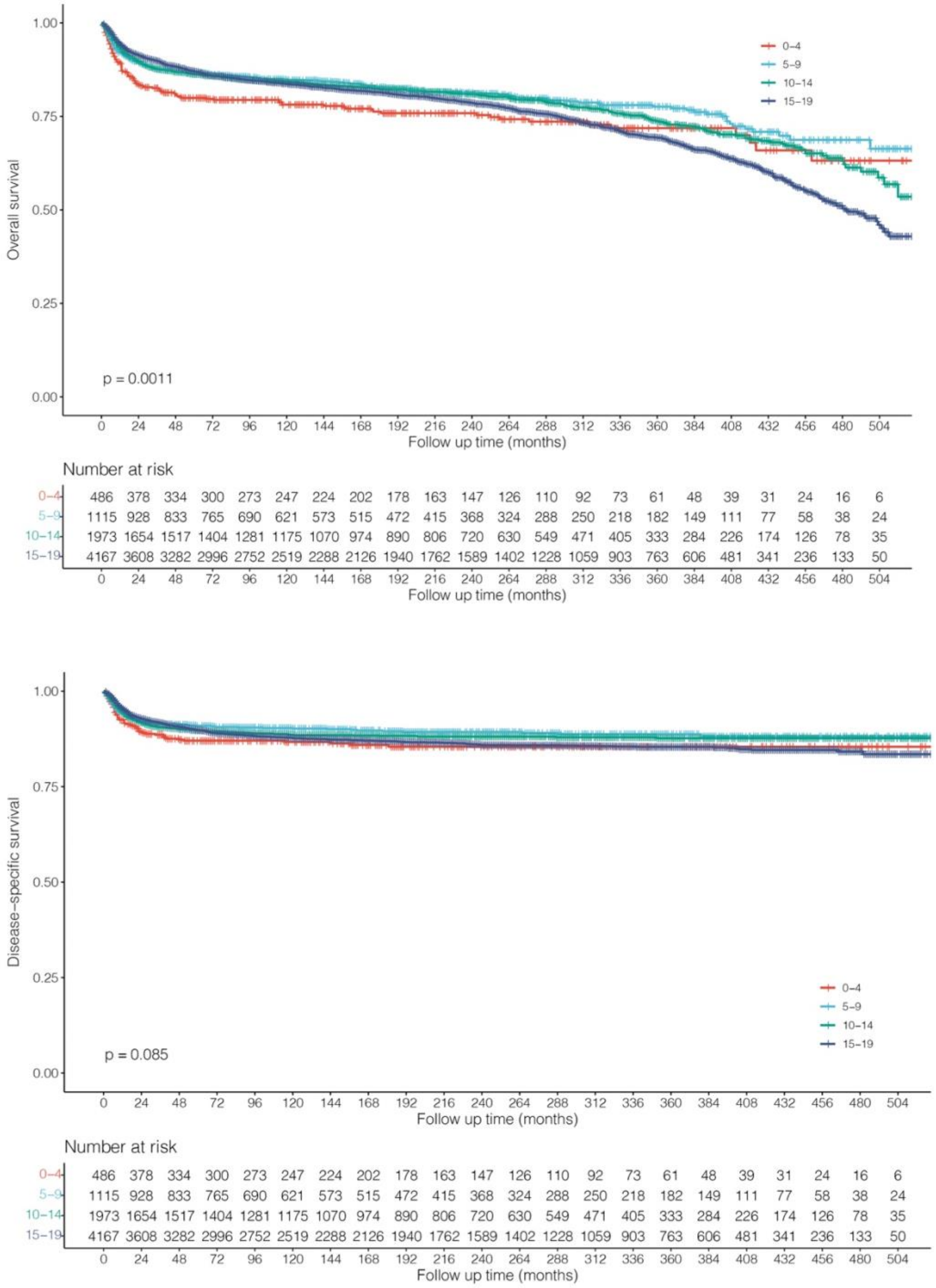

Figure S3. Kaplan–Meier curves for the overall survival and disease-specific survival comparisons between male and female pediatric patient with lymphoma (1975-2018)

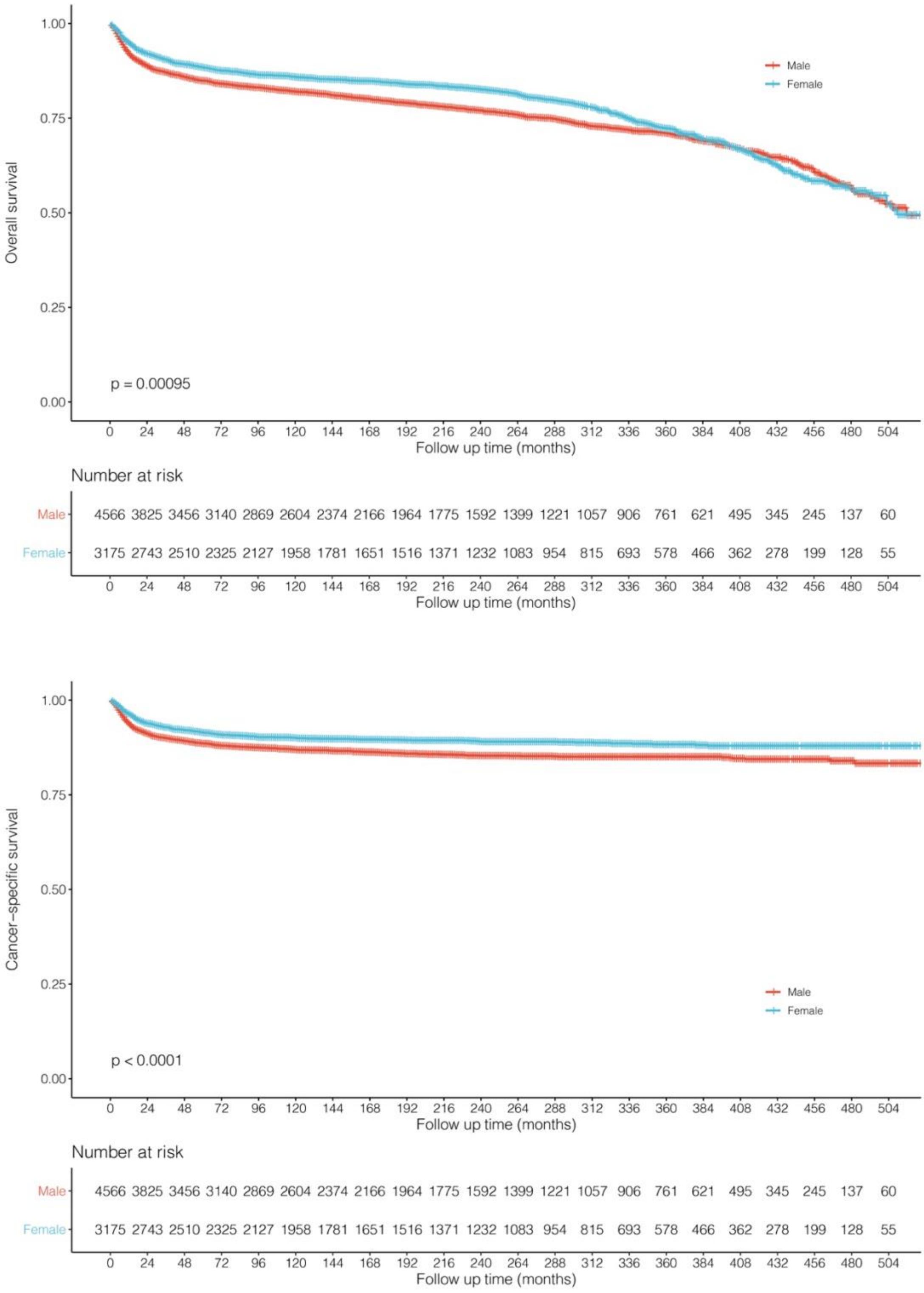

Figure S4. Kaplan–Meier curves for the overall survival and disease-specific survival comparisons among subgroups of pediatric patients with lymphoma by race (1975-2018)

AI/AN/AP, American Indian/Alaska Native/Asian and Pacific Islander.

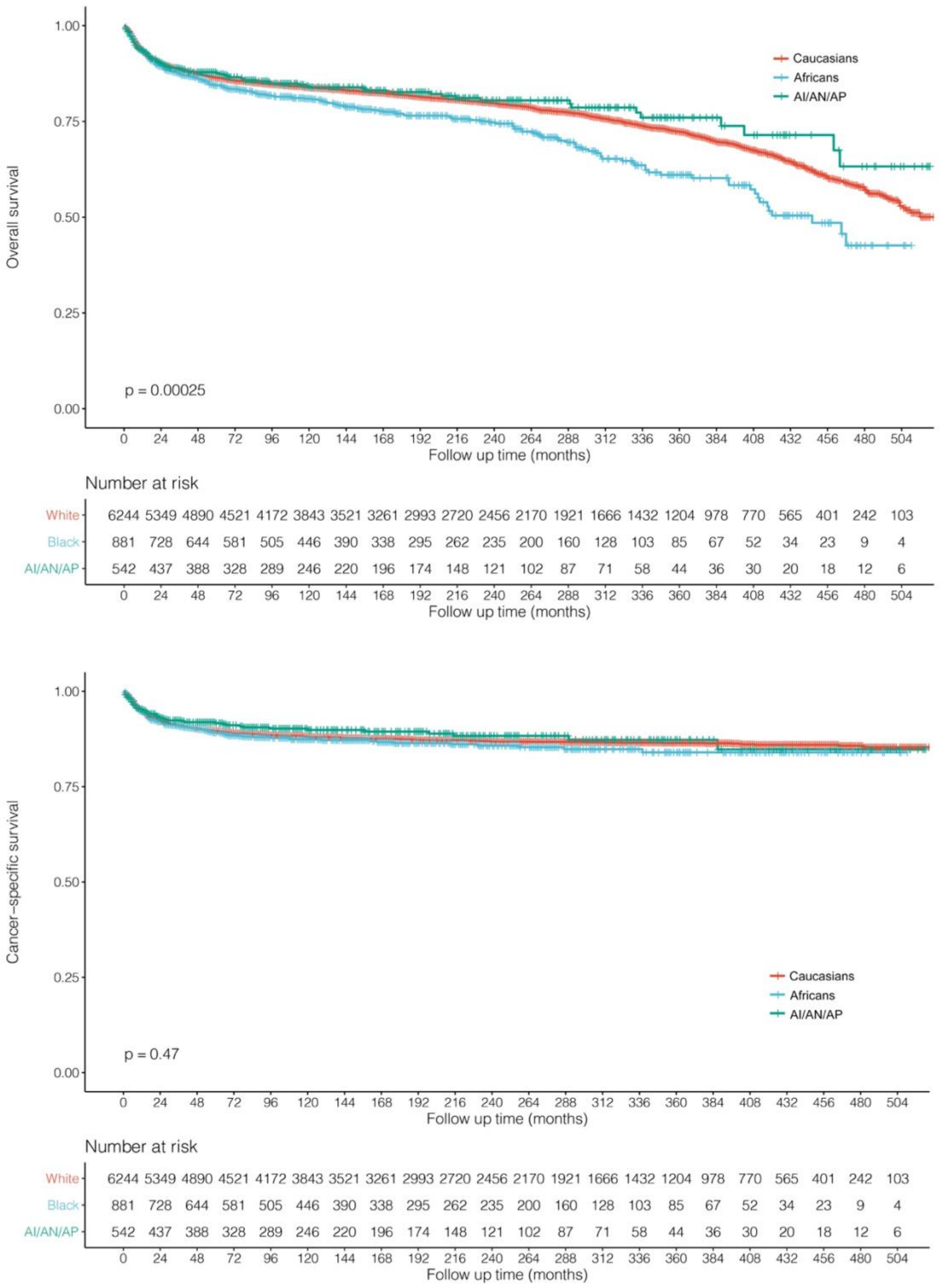

Figure S5. Kaplan–Meier curves for the overall survival and disease-specific survival comparisons between pediatric patients with Hodgkin lymphoma (HL) and Non-Hodgkin lymphoma (NHL) (1975-2018).

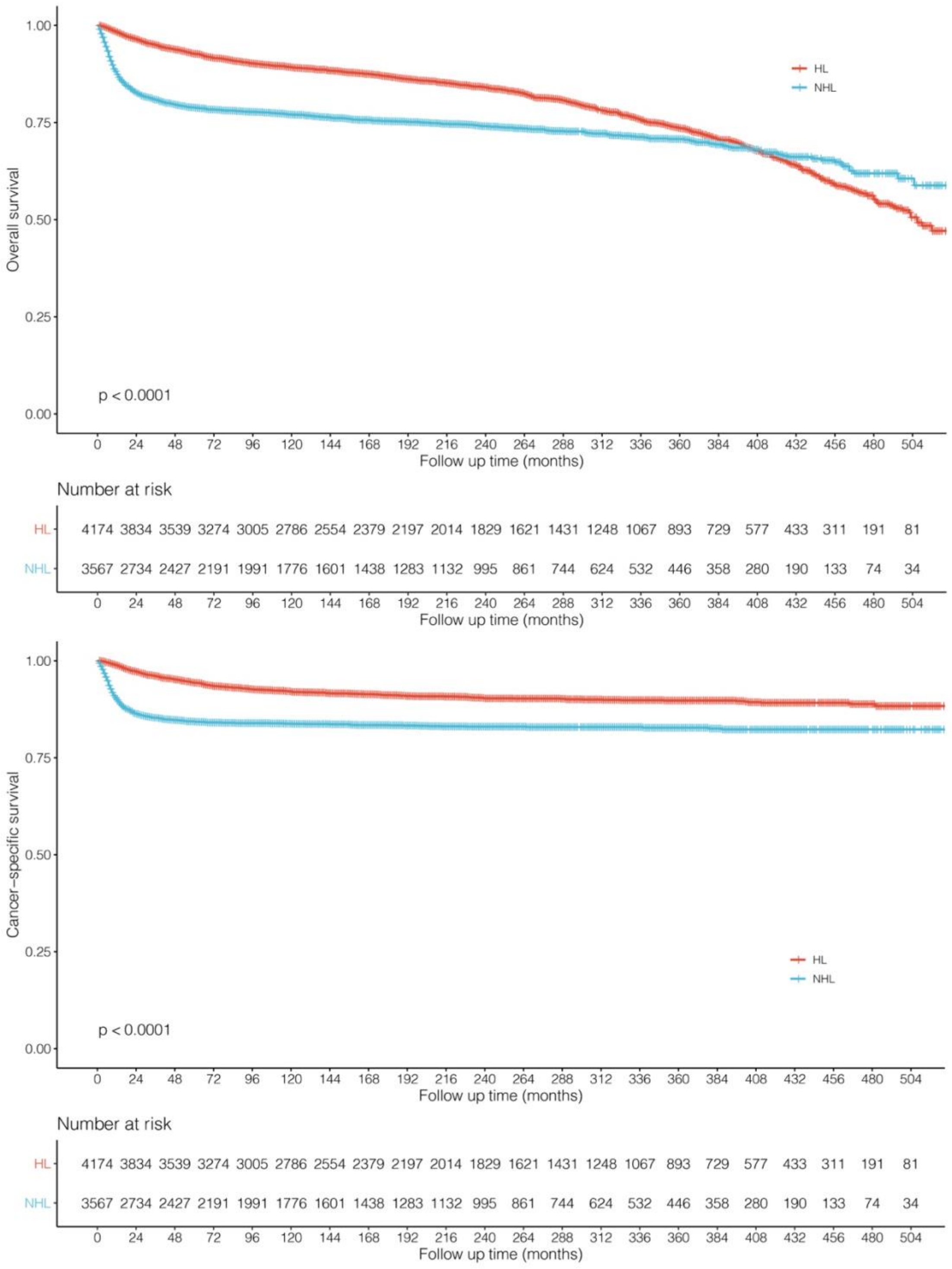

Figure S6. Kaplan–Meier curves for the overall survival and disease-specific survival comparisons among subgroups of pediatric patients with lymphoma by the Ann Arbor stage (1983-2015)

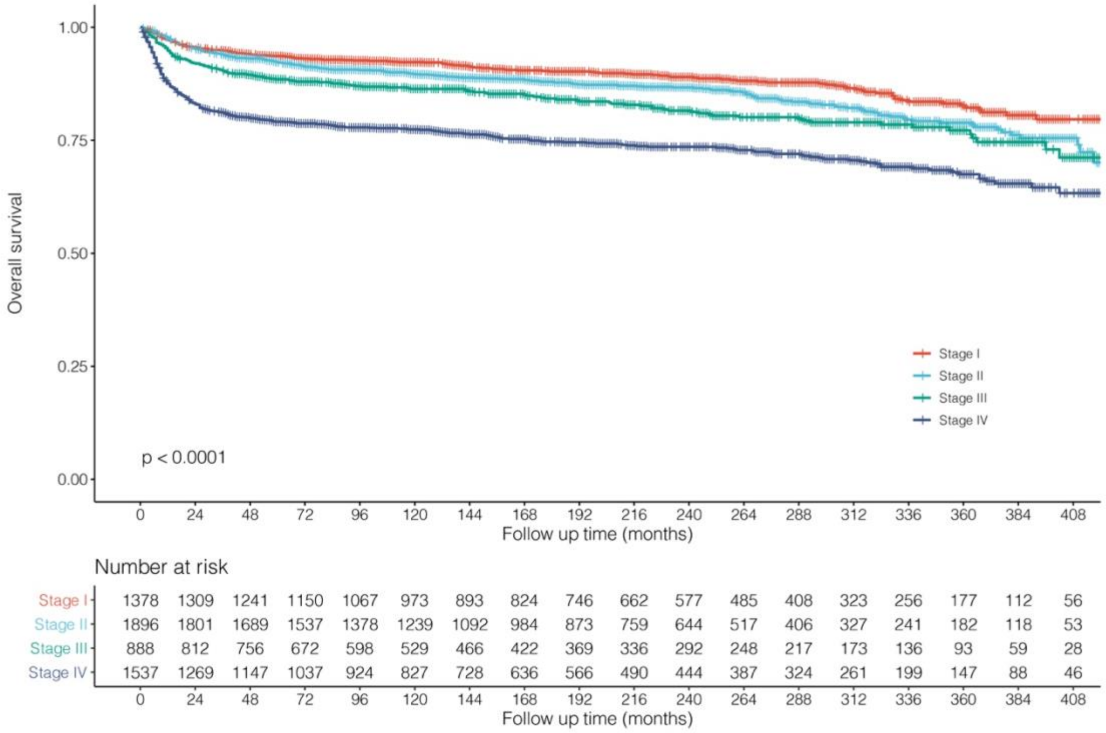

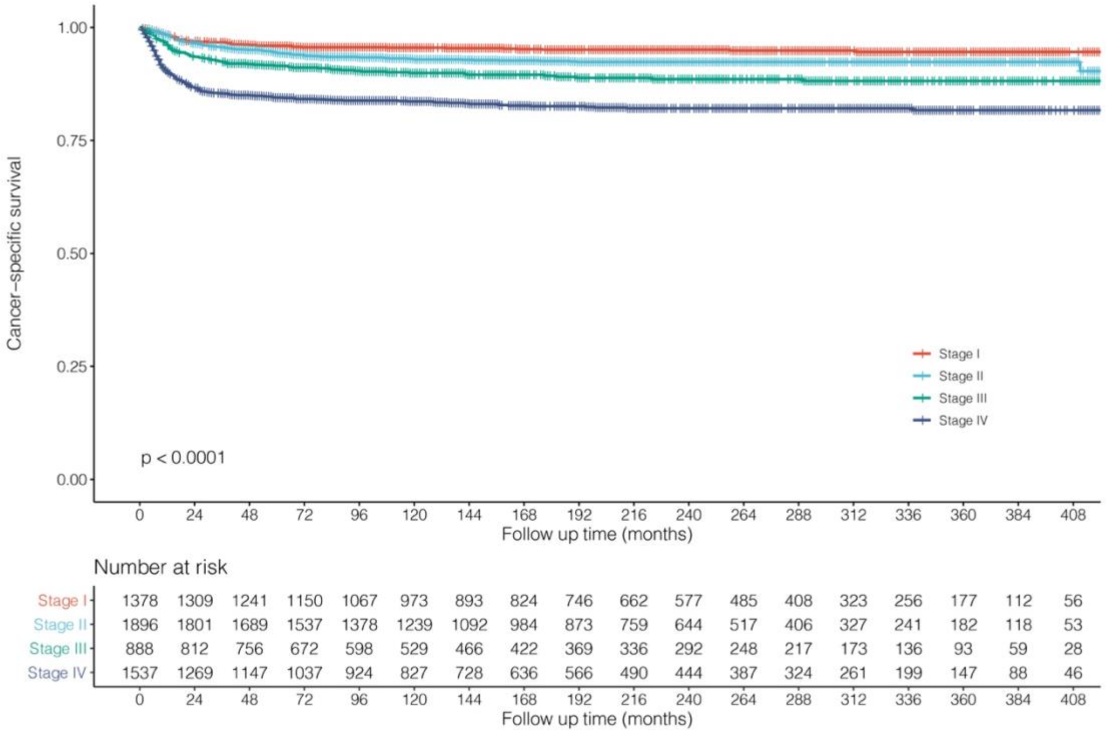

Figure S7. Calibration curves of the nomogram for 1-year, 5-year, and 10-year overall survival (OS) in the training cohort (A) and the validation cohort (B).

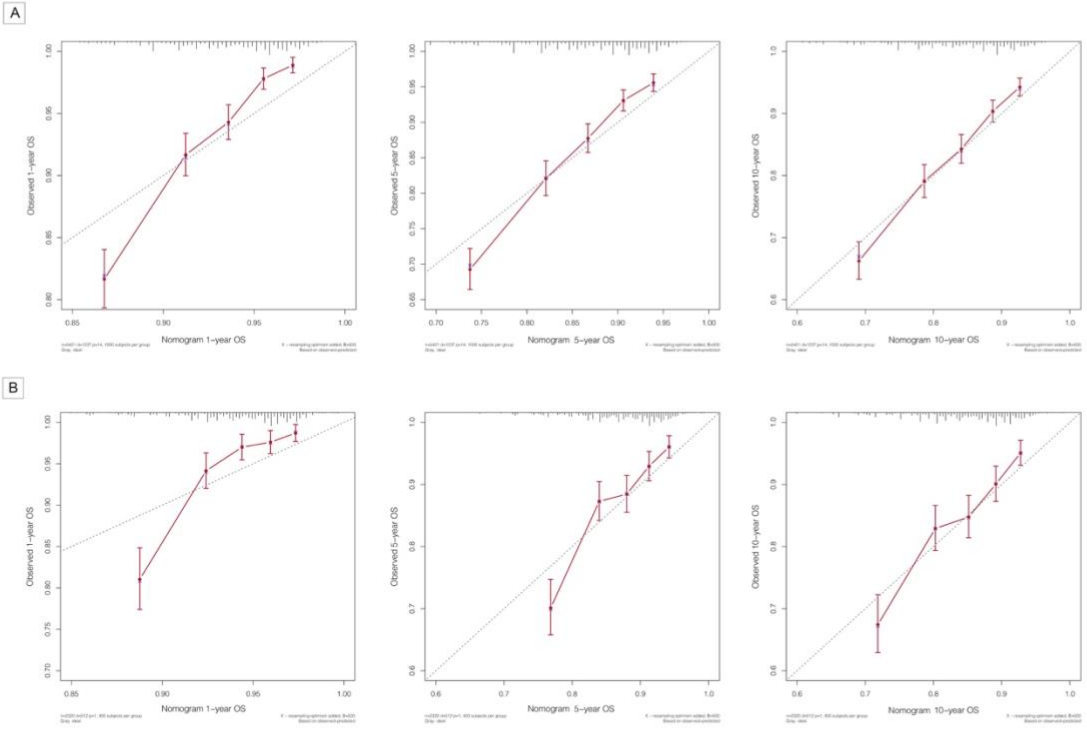

Table S1. Parameters of machine learning algorithms

Algorithm	Parameters
ANN	Number of hidden layers: 3
	Neurons per layer: 64
	Activation function: ReLU
	Optimizer: Adam
	Learning rate: 0.001
	Epochs: 100
	Batch size: 32
XGB	Number of trees: 100
	Learning rate: 0.1
	Max depth: 6
	Subsample: 0.8
	Colsample bytree: 0.8
RFC	Number of trees: 100
	Max depth: None
	Min samples split: 2
	Min samples leaf: 1
	Bootstrap: True
ADB	Number of estimators: 50
	Learning rate: 1.0
	Algorithm: SAMME.R
GBDT	Number of trees: 100
	Learning rate: 0.1
	Max depth: 3
	Min samples split: 2
	Min samples leaf: 1
LR	Penalty: l2
	Solver: liblinear
	C: 1.0

Table S2. Demographic characteristics of pediatric patients with lymphoma.

Characteristic	Count	%
All patients	7871	100
Age, years		
0-4	513	6.5
5-9	1137	14.4
10-14	2006	25.5
15-19	4215	53.6
Sex		
Male	4650	59.1
Female	3221	40.9
Race		
White	6338	80.5
Black	897	11.4
AI/AN/AP	556	7.1
Unknown	80	1.0
Tumor subtype		
N-HL	4144	52.6
E-HL	49	0.6
N-NHL	2543	32.3
E-NHL	1135	14.4
Ann Arbor stage		
I	1388	17.6
II	1901	24.2
III	896	11.4
IV	1394	17.7
Unstaged	2292	29.1
Year of diagnosis		
1975-1979	894	11.4
1980-1984	817	10.4
1985-1989	803	10.2
1990-1994	798	10.1
1995-1999	827	10.5
2000-2004	875	11.1
2005-2009	972	12.3
2010-2014	1051	13.4
2015-2018	834	10.6
Radiotherapy		
None/Unknown	4787	60.8
Yes	3084	39.2

Chemotherapy		
No/Unknown	1708	21.7
Yes	6163	78.3
Vital status		
Alive	5992	76.1
Dead (survival<1 month)	110	1.4
Dead (survival≥1 month)	1749	22.2
Loss to follow-up	20	0.3

AI/AN/AP: American Indian/Alaska Native/Asian and Pacific Islander; HL: Hodgkin lymphoma; N-HL: nodal Hodgkin lymphoma; E-HL: extra-nodal Hodgkin lymphoma; N-NHL: nodal non-Hodgkin lymphoma; E-NHL: extra-nodal non-Hodgkin lymphoma.

Table S3. The 1-year, 5-year, and 10-year overall survival and disease-specific survival rates of pediatric and adult patients with lymphoma

Year of diagnosis	Pediatric patients						Adult patients					
	1y-OS	5y-OS	10y-OS	1y-DSS	5y-DSS	10y-DSS	1y-OS	5y-OS	10y-OS	1y-DSS	5y-DSS	10y-DSS
1975	0.825	0.661	0.598	0.855	0.701	0.655	0.739	0.487	0.346	0.795	0.603	0.5
1976	0.862	0.766	0.706	0.885	0.809	0.77	0.759	0.506	0.356	0.816	0.633	0.541
1977	0.84	0.741	0.679	0.873	0.789	0.75	0.746	0.488	0.35	0.799	0.606	0.513
1978	0.865	0.736	0.685	0.897	0.802	0.771	0.752	0.487	0.338	0.81	0.603	0.507
1979	0.894	0.789	0.728	0.926	0.856	0.813	0.739	0.484	0.36	0.803	0.62	0.547
1980	0.884	0.781	0.74	0.89	0.8	0.779	0.76	0.504	0.363	0.812	0.625	0.543
1981	0.872	0.756	0.711	0.896	0.796	0.782	0.76	0.503	0.357	0.818	0.626	0.533
1982	0.886	0.784	0.747	0.898	0.818	0.786	0.756	0.501	0.364	0.809	0.616	0.535
1983	0.923	0.786	0.738	0.929	0.848	0.822	0.779	0.52	0.38	0.83	0.641	0.557
1984	0.922	0.832	0.778	0.94	0.866	0.822	0.763	0.52	0.373	0.822	0.648	0.558
1985	0.865	0.761	0.735	0.882	0.789	0.776	0.762	0.525	0.384	0.824	0.654	0.568
1986	0.934	0.855	0.849	0.934	0.894	0.887	0.746	0.506	0.363	0.816	0.639	0.553
1987	0.893	0.823	0.804	0.923	0.871	0.85	0.753	0.508	0.369	0.832	0.659	0.572
1988	0.922	0.81	0.791	0.947	0.865	0.851	0.745	0.511	0.374	0.829	0.657	0.571
1989	0.917	0.833	0.803	0.923	0.85	0.826	0.744	0.503	0.365	0.84	0.664	0.579
1990	0.951	0.868	0.833	0.965	0.894	0.887	0.737	0.5	0.367	0.839	0.657	0.57
1991	0.916	0.844	0.832	0.933	0.89	0.89	0.74	0.504	0.365	0.836	0.672	0.588
1992	0.948	0.831	0.825	0.967	0.887	0.88	0.751	0.515	0.388	0.848	0.669	0.592
1993	0.945	0.869	0.848	0.958	0.888	0.881	0.743	0.518	0.389	0.837	0.679	0.607
1994	0.914	0.856	0.845	0.93	0.889	0.877	0.747	0.523	0.393	0.842	0.681	0.605
1995	0.923	0.852	0.838	0.95	0.898	0.891	0.746	0.519	0.395	0.832	0.673	0.603
1996	0.97	0.91	0.898	0.982	0.939	0.932	0.773	0.548	0.416	0.84	0.684	0.618
1997	0.944	0.894	0.883	0.955	0.922	0.916	0.78	0.567	0.427	0.839	0.696	0.626
1998	0.949	0.887	0.859	0.955	0.903	0.897	0.793	0.58	0.445	0.846	0.705	0.643
1999	0.967	0.928	0.908	0.987	0.96	0.953	0.796	0.586	0.447	0.85	0.71	0.646
2000	0.931	0.872	0.862	0.952	0.913	0.913	0.802	0.605	0.473	0.86	0.737	0.68
2001	0.963	0.908	0.889	0.969	0.932	0.932	0.814	0.62	0.487	0.862	0.753	0.694
2002	0.961	0.915	0.887	0.966	0.943	0.926	0.826	0.641	0.511	0.878	0.767	0.716
2003	0.971	0.925	0.919	0.977	0.936	0.93	0.821	0.647	0.511	0.875	0.779	0.73
2004	0.934	0.88	0.861	0.957	0.939	0.926	0.825	0.641	0.504	0.881	0.78	0.728
2005	0.939	0.89	0.878	0.975	0.925	0.925	0.83	0.655	0.508	0.883	0.789	0.735
2006	0.953	0.912	0.9	0.976	0.946	0.934	0.83	0.656	0.519	0.882	0.796	0.744
2007	0.952	0.928	0.898	0.961	0.947	0.942	0.835	0.665	0.528	0.884	0.793	0.754
2008	0.953	0.92	0.89	0.967	0.957	0.942	0.852	0.67	0.535	0.901	0.808	0.765
2009	0.957	0.918	NA	0.971	0.946	NA	0.846	0.675	NA	0.893	0.808	NA
2010	0.944	0.925	NA	0.958	0.943	NA	0.84	0.665	NA	0.886	0.796	NA

2011	0.974	0.953	NA	0.974	0.959	NA	0.843	0.675	NA	0.888	0.807	NA
2012	0.982	0.96	NA	0.987	0.969	NA	0.845	0.678	NA	0.889	0.808	NA
2013	0.961	0.938	NA	0.967	0.949	NA	0.852	0.681	NA	0.897	0.809	NA
2014	0.959	NA	NA	0.968	NA	NA	0.842	NA	NA	0.89	NA	NA
2015	0.972	NA	NA	0.981	NA	NA	0.85	NA	NA	0.895	NA	NA
2016	0.979	NA	NA	0.984	NA	NA	0.855	NA	NA	0.902	NA	NA
2017	0.984	NA	NA	0.994	NA	NA	0.85	NA	NA	0.893	NA	NA

Table S4. Baseline demographic characteristics of the training and validation cohorts of the nomogram.

Characteristic	All	Training cohort	Validation cohort	*P* value
Age, year				
0-4	486	340	146	.57
5-9	1115	764	351	
10-14	1973	1400	573	
15-19	4167	2917	1250	
Sex				
Male	4566	3168	1398	.14
Female	3175	2253	922	
Race				
White	6244	4389	1855	.10
Black	881	620	261	
AI/AN/AP	542	369	173	
Unknown	74	43	31	
Tumor subtype				
HL	4174	2925	1249	.83
N-NHL	2467	1734	733	
E-NHL	1100	762	338	
Ann Arbor stage				
I	1378	956	422	.38
II	1896	1299	597	
III	888	635	253	
IV	1537	1082	455	
Unstaged	2042	1449	593	
Radiotherapy				
No	4663	3245	1418	.30
Yes	3078	2176	902	
Chemotherapy				
No	1622	1121	501	.36
Yes	6119	4300	1819	

AI/AN/AP: American Indian/Alaska Native/Asian and Pacific Islander; HL: Hodgkin lymphoma; NHL: Non-Hodgkin lymphoma; N-NHL: Nodal Non-Hodgkin lymphoma; E-NHL: Extra-nodal Non-Hodgkin lymphoma.
